# Cannabis Use During Early Pregnancy Following Recreational Cannabis Legalization

**DOI:** 10.1001/jamahealthforum.2024.3656

**Published:** 2024-11-01

**Authors:** Kelly C. Young-Wolff, Natalie E. Slama, Lyndsay A. Avalos, Alisa A. Padon, Lynn D. Silver, Sara R. Adams, Monique B. Does, Deborah Ansley, Carley Castellanos, Cynthia I. Campbell, Stacey E. Alexeeff

**Affiliations:** 1 Division of Research, Kaiser Permanente Northern California, Pleasanton; 2 Department of Psychiatry and Behavioral Sciences, University of California, San Francisco; 3 Public Health Institute, Oakland, California; 4 Regional Offices, Kaiser Permanente Northern California, Pleasanton

## Abstract

**Importance:**

It is unknown whether state recreational cannabis legalization (RCL) is related to increased rates of prenatal cannabis use or whether RCL-related changes vary with cannabis screening methods or the local policy environment.

**Objective:**

To test whether RCL in California was associated with changes in prenatal cannabis use rates, whether changes were evident in both self-report and urine toxicology testing, and whether rates varied by local policies banning vs allowing adult-use retailers post-RCL.

**Design, Setting, and Participants:**

This population-based time-series study used data from pregnancies in Kaiser Permanente Northern California universally screened for cannabis use during early pregnancy by self-report and toxicology testing from January 1, 2012, to December 31, 2019. Analyses were conducted from September 2022 to August 2024.

**Exposures:**

California state RCL passage (November 9, 2016) and implementation of legal sales (January 1, 2018) were examined with a 1-month lag. Local policies allowing vs banning medical retailers pre-RCL and adult-use retailers post-RCL were also examined.

**Main Outcomes and Measures:**

Any prenatal cannabis use was based on screening at entrance to prenatal care (typically at 8-10 weeks’ gestation) and defined as (1) a positive urine toxicology test result or self-report, (2) a positive urine toxicology test result, or (3) self-report. Interrupted time series models were fit using Poisson regression, adjusting for age, race and ethnicity, and neighborhood deprivation index.

**Results:**

The sample of 300 993 pregnancies (236 327 unique individuals) comprised 25.9% Asian individuals, 6.4% Black individuals, 26.0% Hispanic individuals, 37.7% White individuals, and 4.1% individuals of other, multiple, or unknown race, with a mean (SD) age of 30.3 (5.4) years. Before RCL implementation, rates of prenatal cannabis use rose steadily from 4.5% in January 2012 to 7.1% in January 2018. There was no change in use rates at the time of RCL passage (level change rate ratio [RR], 1.03; 95% CI, 0.96-1.11) and a statistically significant increase in rates in the first month after RCL implementation, increasing to 8.6% in February 2018 (level change RR, 1.10; 95% CI, 1.04-1.16). Results were similar when defining prenatal cannabis use by (1) a toxicology test or (2) self-report. In local policy analyses, the post-RCL implementation increase in use was only found among those in jurisdictions allowing adult-use cannabis retailers (allowed RR, 1.21; 95% CI, 1.10-1.33; banned RR, 1.01; 95% CI, 0.93-1.10).

**Conclusions and Relevance:**

In this time-series study, RCL implementation in California was associated with an increase in rates of cannabis use during early pregnancy, defined by both self-report and toxicology testing, driven by individuals living in jurisdictions that allowed adult-use retailers.

## Key Points

**Question:**

Did rates of cannabis use during early pregnancy change after recreational cannabis legalization (RCL) in California, and did trends differ in jurisdictions that allowed vs banned adult-use retailers?

**Findings:**

This time-series study of 300 993 pregnancies universally screened for prenatal cannabis use at entrance to prenatal care in Northern California from 2012 to 2019, there was an increase in self-reported and biochemically verified prenatal cannabis use after RCL implementation in 2018. The increase was observed only among those living in jurisdictions allowing adult-use cannabis retailers.

**Meaning:**

The implementation of RCL in California was associated with an increase in prenatal cannabis use, driven by individuals living in jurisdictions that permitted adult-use retailers.

## Teaser

This time-series study tests whether recreational cannabis legalization in California was associated with changes in prenatal cannabis use rates, whether changes were evident in both self-report and urine toxicology testing, and whether rates varied by local policies banning vs allowing adult-use retailers after recreational cannabis legalization.

## Introduction

Prenatal cannabis use is common in the US, and rates are rising.^1,2,3,4^ From 2002 to 2017, the prevalence of self-reported prenatal cannabis use increased from 3% to 7%.^4^ Pregnant individuals report using cannabis to treat mental health and physical symptoms, and some view cannabis as a potentially safer option than prescription medications.^5,6,7^ However, prenatal cannabis use is associated with an increased risk of adverse neonatal outcomes (eg, preterm birth, small for gestational age, low birth weight, neonatal intensive care unit admission) and maternal pregnancy outcomes (eg, gestational hypertension, preeclampsia, placental abruption, weight gain outside of recommendations)^8^ and may also be associated with offspring neurodevelopment.^9,10,11,12,13,14,15^ National medical organizations recommend that clinicians screen for prenatal cannabis use, educate patients about potential risks, and recommend abstinence during pregnancy.^15,16^

Increased rates of prenatal cannabis use may be partially attributable to recreational cannabis legalization (RCL). As of July 2024, 38 states and Washington, DC, have legalized medical cannabis, and 24 states and Washington, DC, have legalized recreational cannabis.^17,18^ California legalized medical cannabis use in 1996 and passed RCL in November 2016. Legal adult-use retail sales began in January 2018 when RCL was fully implemented. Local jurisdictions could ban or allow adult-use retail, and policies varied widely.^19,20^

Epidemiological studies examining RCL and prenatal cannabis use are limited, with conflicting results.^21,22,23,24,25,26,27^ Importantly, while RCL implementation can vary within a state,^28,29,30^ prior studies of state RCL and prenatal cannabis use have not examined variation in local access to cannabis retailers. Furthermore, self-reported use may underestimate the true use prevalence,^31^ and post-RCL increases could reflect an increased willingness to disclose prenatal use post-RCL. RCL studies that examine different screening methods (eg, self-report vs toxicology tests) and include the local policy environment are needed to better guide policy recommendations.

Using 2012 to 2019 data from the Kaiser Permanente Northern California (KPNC) large, health care system with universal screening for cannabis use during early pregnancy, we modeled the rates of prenatal cannabis use pre-RCL passage, post-RCL passage, and after the full RCL implementation that allowed legal adult-use retail sales. We tested whether rates changed in each period, whether changes were evident in both self-report and toxicology testing, and whether rates varied by local policies.

## Methods

### Study Design

KPNC is a large, multispecialty health care system serving approximately 4.6 million diverse members representative of Northern California’s insured population.^32^ All 323 822 pregnancies screened for self-reported prenatal substance use as part of standard prenatal care from January 1, 2012, to December 31, 2019, were eligible for study inclusion.^33^ Pregnancies without a valid address in KPNC’s 35-county catchment area (1248 [0.4%]), missing a response to the self-reported question about prenatal cannabis use (2205 [0.7%]), or missing a urine toxicology test (19 376 [6.0%]) were excluded (eFigure 1 in Supplement 1).

KPNC provided institutional review board approval, with a waiver of patient informed consent and authorization. Study procedures met HIPAA (US Health Insurance Portability and Accountability Act) requirements and 42 CFR part 2 regarding medical records. This study followed the Strengthening the Reporting of Observational Studies in Epidemiology (STROBE) reporting guidelines.

### Measures

#### Prenatal Cannabis Use

The primary outcome was cannabis use during early pregnancy based on universal screening for cannabis at the entrance to prenatal care (typically at approximately 8-10 weeks’ gestation) via both a self-administered questionnaire that assesses any cannabis use since pregnancy (including prenatal use prior to pregnancy recognition) and a urine toxicology test, to which patients provided consent. Confirmatory testing for the presence of the cannabis metabolite, 11-nor-9-carboxy-delta-9-THC, which is detectable for up to about 30 days after last use among those who use regularly, was performed by liquid chromatography–tandem mass spectrometry for all positive immunoassay results (eMethods in Supplement 1). Individuals were classified as having any prenatal use if they self-reported cannabis use since pregnancy or had a positive confirmed toxicology test. Secondary outcomes defined use by (1) a positive urine toxicology test or (2) self-report.

#### RCL

We assessed 2 key time points of RCL: passage and implementation. California voters passed RCL on November 9, 2016.^34^ After RCL passage, possession and home grows became legal immediately and nonmedical use was further decriminalized. RCL was fully implemented on January 1, 2018, when legal adult-use sales began and adult-use retailers opened.

#### Local Cannabis Storefront Retail Policy

We assessed local cannabis storefront retail policy for medical and adult-use retailers. Local retailer laws (banned vs allowed) were extracted from the CannaRegs (Fyllo) commercial regulatory database, complemented by verification on jurisdictions’ (city or county) websites and municipal codes, as well as outreach to city or county staff when needed.^19^

Each pregnancy was categorized based on local laws for the jurisdiction where the patient resided at the time of the toxicology test. During the pre-RCL implementation period, pregnancies were categorized based on whether individuals lived in a jurisdiction that allowed medical retail any time during 2017 vs banned medical retail that year. During the post-RCL implementation period, we incorporated time-varying changes in each jurisdiction’s policy based on the policy at the time of the toxicology test. Pregnancies were categorized based on whether individuals lived in jurisdictions that (1) allowed vs banned adult-use retail and (2) allowed any retail (medical or adult use) vs banned all retail.

#### Sociodemographics

Electronic health records provided data on patients’ age (<25, 25 to <35, and ≥35 years), neighborhood deprivation index (NDI^35^; quartiles or missing), and geocoded home addresses within 90 days of the toxicology test. We also included data on self-reported race and ethnicity (Asian, Black, White, or other [including Alaska Native, American Indian, Hawaiian Islander, and Pacific Islander]/multiracial/unknown; Hispanic or non-Hispanic) as a social construct due to known differences in the prevalence of prenatal cannabis use by race and ethnicity and evidence of inequities in testing for prenatal substance use in health care systems without universal screening.^36^

### Statistical Analysis

Monthly cannabis use rates were standardized to the age, race and ethnicity, and NDI distribution of the sample during 2019 to account for any differences in distributions over time.^37^ We conducted interrupted time series analyses with segmented Poisson regression models accounting for overdispersion using the quasi-Poisson family.^38^ The outcome was the monthly count of pregnancies positive for cannabis use, and the offset was the log of the standardized population that month. We reported rate ratios (RRs) and 95% CIs.

We tested for interruptions to the time series at RCL (1) passage in 2016 and (2) implementation in 2018 (eMethods in Supplement 1). We tested for immediate level and slope changes for the primary and secondary outcomes. A 1-month lag in outcomes was used to reflect the timing of data collection because patients self-report any cannabis use since the start of pregnancy, and toxicology tests reflect use up to approximately 30 days prior. Thus, the period pre-RCL passage included pregnancies screened January 2012 to November 2016, the period post-RCL passage and pre-RCL implementation included pregnancies screened December 2016 to January 2018, and the period post-RCL implementation included pregnancies screened February 2018 to December 2019. Preliminary models assessed the linearity of trends before RCL passage by adding a quadratic term for time and confirmed that the trend was linear (all *P* > .10; eTable 1 in Supplement 1). Sensitivity analyses assessed whether post-RCL implementation level and slope changes were sensitive to the data point in the first month after legal sales by modeling a separate level change for that month and estimating the remaining post-RCL implementation level and slope changes without that month. We also conducted sensitivity analyses with seasonal adjustment using Fourier terms.^39^

Local policies were examined in several ways. First, we fit separate models stratified by the pre-RCL implementation medical retail policy (banned vs allowed), followed by tests for statistically significant differences in a combined model using interaction terms. Second, we fit a combined model with separate intercepts and slopes for jurisdictions allowing vs banning medical storefront retail in the pre-RCL implementation period, as well as separate level and slope changes for jurisdictions allowing vs banning adult-use storefront retail in the post-RCL implementation period. We tested for differences using interaction terms.

In sensitivity analyses of local policy differences post-RCL implementation, we examined slope and level changes in jurisdictions allowing any retail (medical or adult use) vs jurisdictions banning all retail. In additional sensitivity analyses, we fit models stratified by the pre-RCL implementation medical retail policy, with separate level and slope changes after RCL implementation based on the adult-use policy of each jurisdiction at the time of the pregnancy, noting that the interpretation of this analysis is limited because only 1 jurisdiction that had previously banned medical retail switched to a policy allowing adult-use retail at the time of RCL implementation in January 2018.

We also conducted sensitivity analyses to evaluate the potential effect of unmeasured confounding on the results by computing e-values.^40,41^ Standardized difference was calculated as the difference in proportions divided by the standard error.^42^

All analyses were conducted in SAS, version 9.4 (SAS Institute), and R, version 4.0.2 (R Project for Statistical Computing), from September 2022 to August 2024. Two-sided *P* < .05 was considered statistically significant.

## Results

### Sample Characteristics

The sample of 300 993 pregnancies (236 327 unique individuals) comprised 25.9% Asian individuals, 6.4% Black individuals, 26.0% Hispanic individuals, 37.7% White individuals, and 4.1% individuals of other, multiple, or unknown race, with a mean (SD) age of 30.3 (5.4) years and gestational age at toxicology test of 8.9 (4.8) weeks (Table). Overall, 6.6% screened positive for cannabis use (self-report or toxicology test; 1.1% were positive only via self-report, 3.6% were positive only via toxicology, and 1.9% were positive from both toxicology and self-report). The sample included 173 906 pregnancies (57.8%) pre-RCL passage, 47 450 pregnancies (15.8%) post-RCL passage and pre-RCL implementation, and 79 637 pregnancies (26.5%) post-RCL implementation. There were negligible differences in age, race and ethnicity, NDI, and gestational age (1) among those included vs excluded due to missing data on prenatal cannabis use and (2) between the 3 time periods (standardized differences, <0.2; eTable 2 in Supplement 1 and Table).^33,42^

**Table.** Baseline Characteristics of Pregnancies Screened for Prenatal Cannabis Use Between January 1, 2012, and December 31, 2019

Baseline characteristics	No. (%)^a^				Standardized difference^c^
	Overall (N = 300 993)	Pre-RCL passage (n = 173 906)^b^	Post-RCL passage, pre-RCL implementation (n = 47 450)^b^	Post-RCL implementation (n = 79 637)^b^	
Age at pregnancy onset, y					
<25	44 437 (14.7)	28 176 (16.2)	6397 (13.5)	9864 (12.4)	0.10
25 to <35	189 259 (62.9)	109 021 (62.7)	30 071 (63.4)	50 167 (63.0)	
≥35	67 297 (22.4)	36 709 (21.1)	10 982 (23.1)	19 606 (24.6)	
Race and ethnicity^d^					
Hispanic	78 304 (26.0)	44 536 (25.6)	12 365 (26.1)	21 403 (26.9)	0.03
Non-Hispanic					
Asian	77 799 (25.9)	44 130 (25.4)	12 418 (26.2)	21 251 (26.7)	
Black	19 330 (6.4)	11 261 (6.5)	3008 (6.3)	5061 (6.4)	
White	113 324 (37.6)	67 227 (38.7)	17 672 (37.2)	28 425 (35.7)	
Other/multiracial/unknown	12 236 (4.1)	6752 (3.9)	1987 (4.2)	3497 (4.4)	
Gestational age at urine toxicology, mean (SD), wk	8.9 (4.8)	9.3 (4.8)	8.8 (5.0)	8.1 (4.6)	0.10
Neighborhood deprivation index					
Quartile 1 (least deprived)	71 667 (23.8)	39 020 (22.4)	11 181 (23.6)	21 466 (27.0)	0.06
Quartile 2	71 050 (23.6)	42 383 (24.4)	11 232 (23.7)	17 435 (21.9)	
Quartile 3	70 793 (23.5)	41 121 (23.6)	10 915 (23.0)	18 757 (23.6)	
Quartile 4 (most deprived)	71 598 (23.8)	41 351 (23.8)	11 422 (24.1)	18 825 (23.6)	
Missing	15 885 (5.3)	10 031 (5.8)	2700 (5.7)	3154 (4.0)	

Abbreviation: RCL, recreational cannabis legalization.

^a^ Percentages may not add to 100 due to rounding.

^b^ A 1-month lag was used to reflect the timing of data collection because self-report asks about use since the start of pregnancy and toxicology tests reflect use up to 30 days prior. Thus, the period pre-RCL passage included pregnancies screened January 1, 2012, to November 30, 2016; the period post-RCL passage and pre-RCL implementation included pregnancies screened December 1, 2016, to January 31, 2018; and the period post-RCL implementation included pregnancies screened February 1, 2018, to December 31, 2019.

^c^ Standardized difference is the difference in means or proportions divided by standard error; imbalance is defined as an absolute value greater than 0.20 (small effect size).^33^

^d^ Race and ethnicity were self-reported by individuals. Other race includes Alaska Native, American Indian, Hawaiian Islander, and Pacific Islander. These categories were grouped together owing to small sample sizes.

### RCL Passage

**Figure 1.**
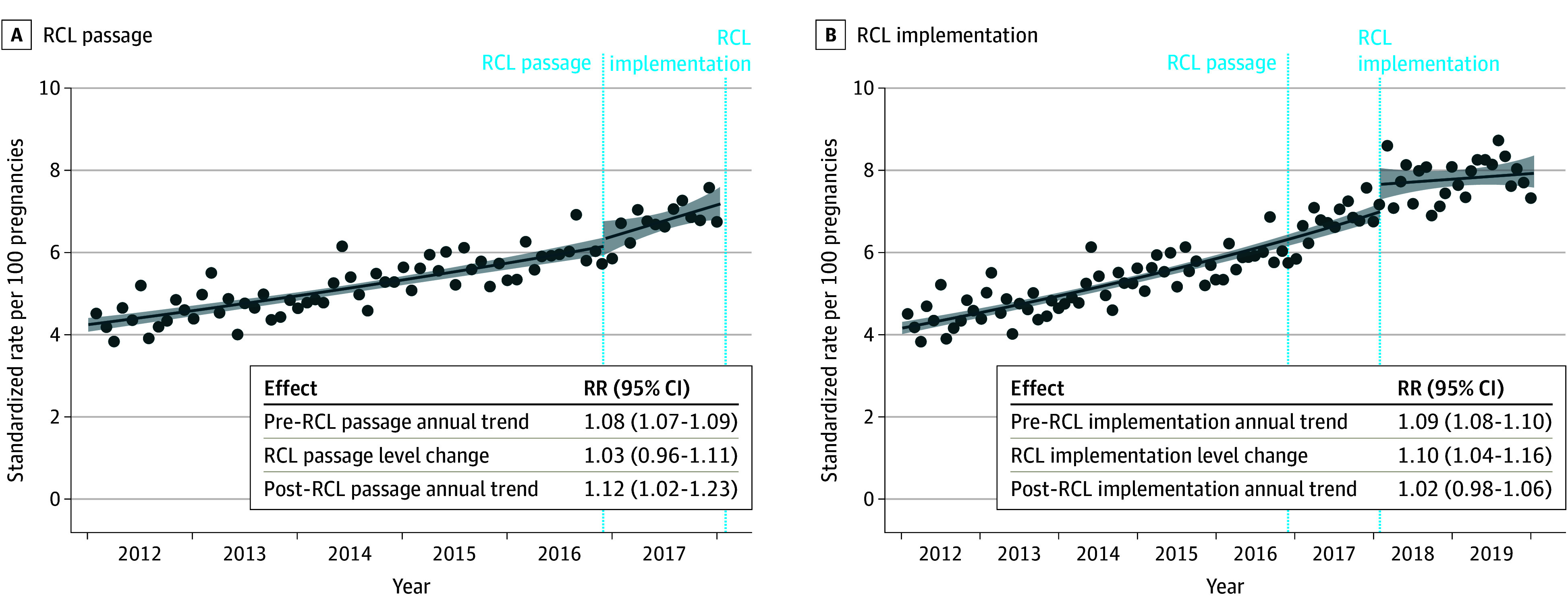
Changes in Prenatal Cannabis Use by Self-Report or Toxicology Testing Before and After Recreational Cannabis Legalization (RCL) Passage and Implementation Relative rates (RRs) are standardized to age, race and ethnicity, and neighborhood deprivation index of the pregnancies in the overall study sample during 2019. The lines show the fitted regression of the interrupted time series model, the dots show the monthly standardized rates, and the shaded areas represent the 95% CIs of the fitted regression line. Prenatal cannabis use was based on a positive urine toxicology test or self-report conducted as part of standard prenatal care (typically around 8-10 weeks’ gestation).

Changes in the rate of prenatal cannabis use at the time of RCL passage were tested first. Before RCL passage, rates of prenatal cannabis use increased steadily from 4.5% in January 2012 to 5.8% in December 2016 (annual trend, 1.08; 95% CI, 1.07-1.09; Figure 1A and eTable 1 in Supplement 1). There was no level change or slope change in the rate of prenatal cannabis use at the time of RCL passage in 2016 (Figure 1A and eTable 3 in Supplement 1).

### RCL Implementation

Based on the findings of no interruption to the time series at the time of RCL passage, all subsequent models modeled the entire pre-RCL implementation period (January 2012-January 2018) using a linear trend. Before RCL implementation, prenatal cannabis use rates rose from 4.5% in January 2012 to 7.1% in January 2018, rising steadily at an annual relative rate of 1.09 (95% CI, 1.08-1.10) (Figure 1B). There was a statistically significant increase in rates of use after RCL implementation (level change RR, 1.10; 95% CI, 1.04-1.16). There was also a slope change after RCL implementation, where annual rates flattened out (post-RCL slope RR, 1.02; 95% CI, 0.98-1.06; slope change *P* = .004), with an average rate of cannabis use of 7.8% from February 2018 to December 2019. In the first month post-RCL implementation, the rate of prenatal cannabis use peaked at 8.6% (level change RR, 1.22; 95% CI, 1.09-1.36). The post-RCL implementation level and slope changes were similar in sensitivity analyses without that month included and in sensitivity analyses with seasonal adjustment using Fourier terms (eTable 4 in Supplement 1).

Results for prenatal cannabis use defined by a toxicology test or by self-report followed a similar pattern. Pre-RCL implementation, rates increased steadily (toxicology annual RR, 1.09; 95% CI, 1.08-1.10; self-report annual RR, 1.09; 95% CI, 1.07-1.10). There was a statistically significant increase post-RCL implementation vs pre-RCL implementation (toxicology level change RR, 1.07; 95% CI, 1.01-1.14; self-report level change RR, 1.21; 95% CI, 1.11-1.32). Post-RCL implementation rates flattened out (toxicology annual RR, 0.99; 95% CI, 0.95-1.04; self-report annual RR, 1.05; 95% CI, 0.99-1.12; eFigure 2 in Supplement 1).

### Medical Storefront Retail Policy Before RCL Implementation

**Figure 2.**
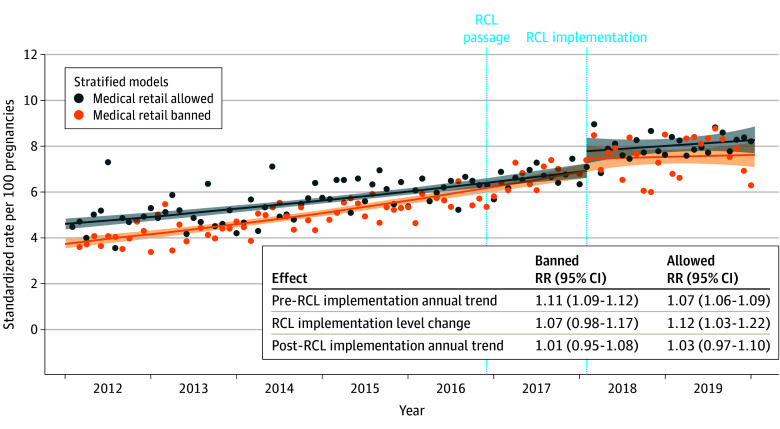
Changes in Prenatal Cannabis Use by Self-Report or Urine Toxicology Before and After Recreational Cannabis Legalization (RCL) Implementation, Stratified by the Pre-RCL Medical Storefront Retail Policy Relative rates (RRs) are standardized to age, race and ethnicity, and neighborhood deprivation index of the pregnancies in the overall study sample during 2019. The lines show the fitted regression of the interrupted time series model, the dots show the monthly standardized rates, and the shaded areas represent the 95% CIs of the fitted regression line. Prenatal cannabis use was based on a positive urine toxicology test or self-report conducted as part of standard prenatal care (typically around 8-10 weeks’ gestation).

Pre-RCL implementation, when only medical cannabis retail was legal in California, 190 jurisdictions banned storefront retailers (166 cities; 24 counties), and most of the sample (56.1%) lived in those jurisdictions. Fifty-four jurisdictions allowed storefront retailers (43 cities; 11 counties). Figure 2 shows rates of prenatal cannabis use before and after RCL implementation, stratified by the pre-RCL medical storefront retail policy. Before RCL implementation, rates of cannabis use differed in jurisdictions that banned vs allowed medical retailers, with the most pronounced difference at the start of the study in 2012 (allowed intercept, 4.6%; banned intercept, 3.8%; intercept RR, 1.23; 95% CI, 1.14-1.32; Figure 2). Rates of prenatal cannabis use rose steadily in jurisdictions allowing medical retail (annual RR, 1.07; 95% CI, 1.06-1.09; Figure 2) but rose even faster in jurisdictions banning medical retail (annual RR, 1.11; 95% CI, 1.09-1.12; interaction *P* < .001). Interestingly, the 2 groups converged to the same rate just before RCL implementation, with the trend lines reaching an average rate of prenatal cannabis use of 7.3% in the month before RCL implementation (Figure 2). Immediately post-RCL implementation vs pre-RCL implementation, rates of prenatal cannabis use increased among pregnancies in jurisdictions that allowed medical retail pre-RCL (level change RR, 1.12; 95% CI, 1.03-1.22), but there was not a statistically significant increase among pregnancies in jurisdictions that banned medical retail (level change RR, 1.07; 95% CI, 0.98-1.17; Figure 2 and eTable 5 in Supplement 1). Annual rates then leveled off post-RCL implementation in both groups (Figure 2).

### Adult-Use Storefront Retail Policy After RCL Implementation

**Figure 3.**
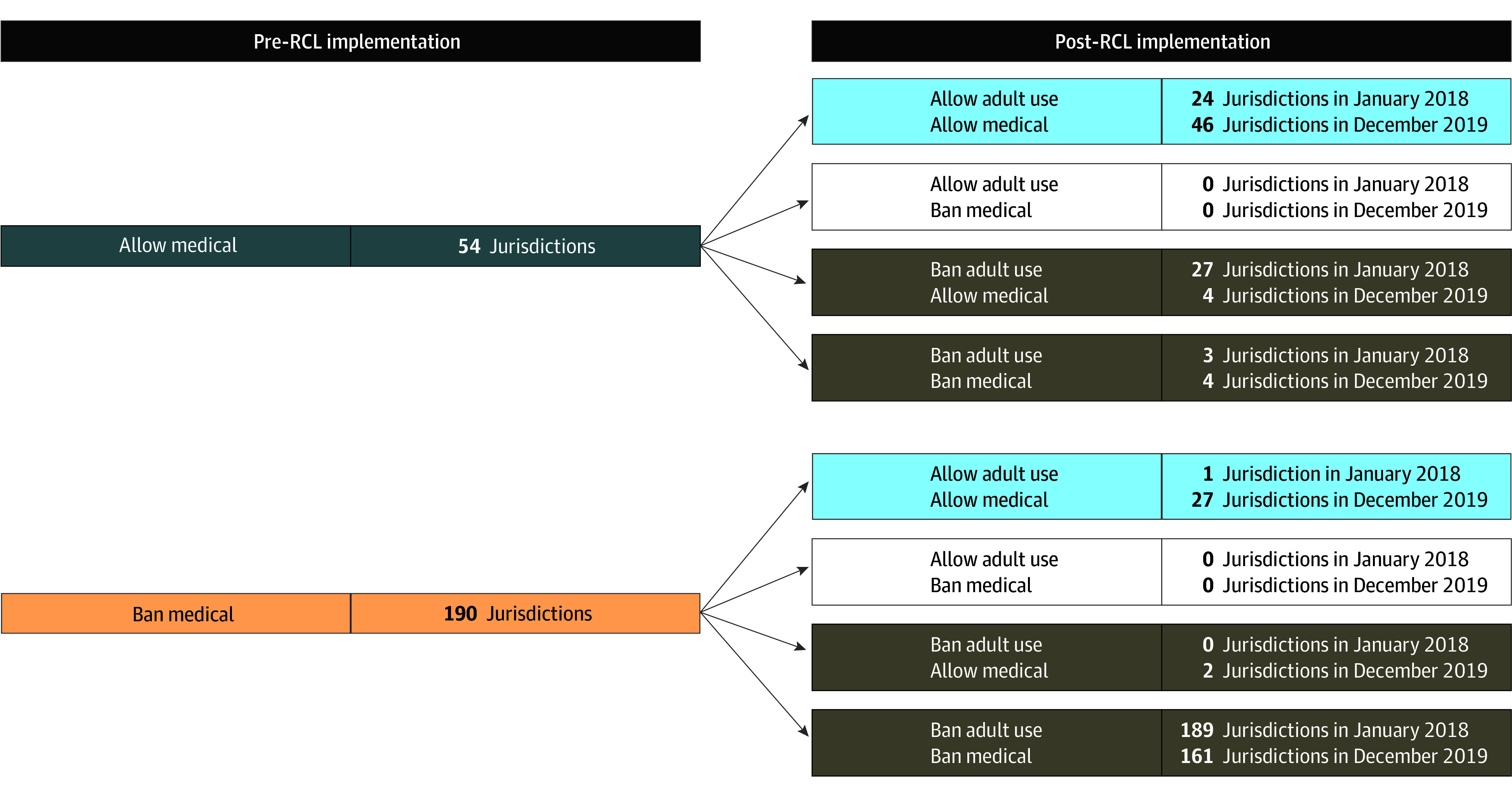
Categorization of Storefront Retail Policy Indicating the Pre–Recreational Cannabis Legalization (RCL) Medical Policy and Post-RCL Adult-Use Policy In the period pre-RCL implementation, pregnancies were grouped based on whether medical retail was allowed or banned in individuals’ jurisdictions. In the period post-RCL implementation, pregnancies were grouped based on whether adult-use retail was allowed or banned in each jurisdiction at the time of screening (corresponds to analyses in Figure 2). Initially, 217 of the 244 jurisdictions (88.9%) had the same policy for medical as for adult-use storefront retailers (eg, both allowed or both banned). By the end of the study period, 51 jurisdictions (20.9%) changed policies and 238 jurisdictions (97.5%) enacted the same policy for medical and adult-use storefront retailers.

After RCL implementation, adult-use storefront retailers were allowed to open. Post-RCL implementation, 50.3% of the study sample lived in jurisdictions that banned medical and adult-use retailers, 5.6% lived in jurisdictions that allowed medical only, and 44.1% lived in jurisdictions that allowed both. Figure 3 shows the complexity of the combinations of policies adopted and the changes in policies during the post-RCL implementation period. For example, of the 54 jurisdictions that had allowed storefront medical retailers before RCL implementation, 24 of them allowed both adult use and medical use after RCL implementation began in 2018, 27 of them banned adult use but continued to allow medical use, and 3 jurisdictions decided to switch policies to ban both adult use and medical use.

**Figure 4.**
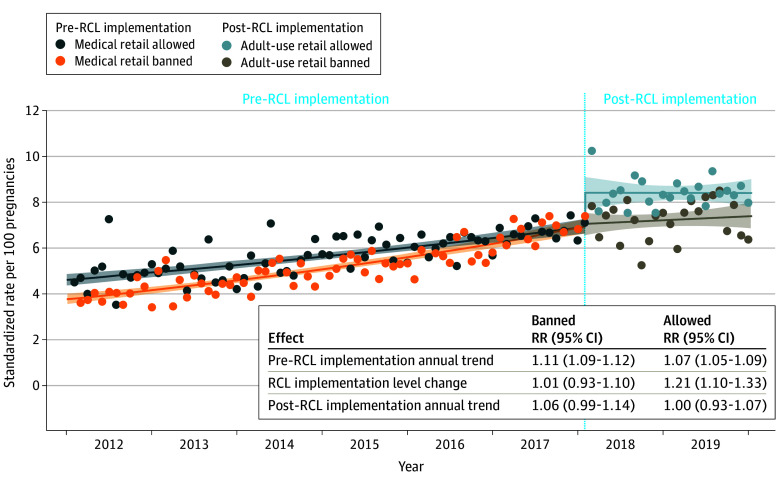
Changes in Prenatal Cannabis Use by Self-Report or Toxicology Testing, Accounting for the Medical Retail Policy in the Pre–Recreational Cannabis Legalization (RCL) Implementation Period and the Adult-Use Retail Policy in the Post-RCL Implementation Period Relative rates (RRs) are standardized to age, race and ethnicity, and neighborhood deprivation index of the pregnancies in the overall study sample during 2019. The lines show the fitted regression of the interrupted time series model, the dots show the monthly standardized rates, and the shaded areas represent the 95% CIs of the fitted regression line. Prenatal cannabis use was based on a positive urine toxicology test or self-report conducted as part of standard prenatal care (typically around 8-10 weeks’ gestation).

The association of RCL implementation with prenatal cannabis use differed by local adult-use retail policy (Figure 4). There was a statistically significant increase in rates immediately post-RCL implementation vs pre-RCL implementation in jurisdictions allowing adult-use retail (level change RR, 1.21; 95% CI, 1.10-1.33) but not in jurisdictions banning adult-use retail (level change RR, 1.01; 95% CI, 0.93-1.10; interaction *P* = .001; Figure 4). Post-RCL implementation, annual rates flattened out in both groups (allowed adult-use retail annual RR, 1.00; 95% CI, 0.93-1.07; banned adult-use retail annual RR, 1.06; 95% CI, 0.99-1.14). At study end (December 2019), average rates remained higher in jurisdictions that allowed (8.4%) vs banned (7.4%) adult-use retail (Figure 4). Results were generally similar in sensitivity analyses that estimated the post-RCL implementation level and slope changes without the first month post-RCL implementation, as well as in sensitivity analyses that examined any post-RCL cannabis retail allowed (medical or adult use) vs all cannabis use retail banned (eTable 5 and eFigures 3 and 4 in Supplement 1).

In sensitivity analyses stratified by medical storefront retail policy in place pre-RCL implementation, results were generally similar to the primary analyses (eFigure 5 in Supplement 1). Immediately post-RCL implementation vs pre-RCL implementation, rates of use increased in jurisdictions allowing adult-use retail (level changes: medical allowed pre-RCL implementation RR, 1.18; 95% CI, 1.06-1.32; medical banned pre-RCL implementation RR, 1.31; 95% CI, 0.96-1.80). There was no increase in cannabis use within jurisdictions banning adult-use retail (level changes: medical allowed pre-RCL implementation RR, 1.00; 95% CI, 0.83-1.22; medical banned pre-RCL implementation RR, 0.10; 95% CI, 0.90-1.01). Although the level change for allowing adult-use retail did not reach statistical significance among the group that had previously banned medical retail, at the time of implementation in January 2018 this included only 1 jurisdiction (Figure 3), which is why the combined model shown in Figure 4 is the primary analysis.

### E-Value Sensitivity Analyses for Unmeasured Confounding

The e-value for the post-RCL implementation-level change RR was 1.423, meaning that an unmeasured confounder would need to have associations of at least that magnitude with both the exposure and the outcome to fully explain the reported RR for the post-RCL implementation level change (eTable 6 in Supplement 1). Since the exposure is the time of the interruption, the unmeasured confounder would need to be associated with the time of post-RCL implementation. If the unmeasured confounder had a weaker association than 1.423 with the time of post-RCL implementation, the unmeasured confounder would need an association with cannabis use that was stronger than 1.423 to fully explain the estimated association for the post-RCL implementation level change.

## Discussion

In this population-based interrupted time-series study of 300 993 pregnancies universally screened for cannabis use during early pregnancy in a large integrated health care system in Northern California from 2012 to 2019, there was a level change in prenatal cannabis use after RCL implementation when adult-use retailers opened. Furthermore, while rates of cannabis use were higher based on urine toxicology testing vs self-report,^31^ the level change after RCL implementation occurred regardless of the cannabis screening method, indicating that results were not solely associated with a greater willingness to disclose use post-RCL implementation. Steady increases were seen during the period of progressive decriminalization pre-RCL passage, but the level change in prenatal cannabis use only occurred after RCL implementation when retailers opened and not immediately post-RCL passage before legal adult-use sales began.

While rates of prenatal cannabis use were initially higher in jurisdictions that allowed vs banned medical retailers, rates increased faster in jurisdictions with medical retail bans. Rates for both groups of pregnancies converged before RCL implementation. The level change in use post-RCL implementation occurred only in jurisdictions allowing adult-use retailers, highlighting the importance of accounting for heterogeneity in the local implementation of state-level policy. However, the post-RCL implementation slope was flattened for jurisdictions allowing adult-use retailers, and it is possible that the changes observed post-RCL implementation could be time limited.

A recent systematic review indicated that while cannabis legalization might increase cannabis use during the preconception, prenatal, and postpartum periods, existing research is subject to bias due to confounding, selection, and misclassification.^25^ Epidemiological studies examining the effect of state RCL on prenatal cannabis use and cannabis-related fetal and neonatal outcomes are limited, with conflicting results, and have not examined variations in local policy.^21,22,23,24,25,26,27^ Importantly, the present results suggest that RCL implementation was only associated with a level change in prenatal cannabis use among pregnant individuals in jurisdictions that allowed adult-use retailers. However, we cannot randomize jurisdiction policies, and these findings have inherent uncertainty.

Results extend our prior work showing that pregnant individuals with a shorter drive time to the nearest retailer or a greater number of retailers within a 15-minute drive from their homes had higher odds of prenatal cannabis use during the first year after RCL implementation.^43^ Furthermore, qualitative data indicate that pregnant individuals in Northern California who used cannabis perceived that prenatal cannabis use increased post-RCL in California in part due to increased access to cannabis, advertising, and marketing.^44^ Notably, in states with RCL, pregnant individuals report seeking advice and information around prenatal cannabis use from cannabis retailer employees,^44,45^ who may advise that prenatal use is safe and effective for treating pregnancy-related symptoms.^46^ Additional research is needed to better understand the mechanisms through which allowing cannabis retail sales might be associated with prenatal cannabis use, to guide the development of more tailored interventions to offset RCL-related increases in prenatal cannabis use.

Clinicians can provide education to all patients during the preconception and prenatal periods about the potential risks associated with prenatal cannabis use so that patients can make well-informed decisions. Importantly, while California does not consider prenatal cannabis use to be child abuse, any RCL-related increases in use may have particularly severe consequences for families living in the many states with punitive policies around prenatal substance use. Research is needed to examine whether RCL exacerbates existing disparities in prenatal cannabis use, cannabis-related social consequences, or repercussions for honest disclosure of use.^47^

### Strengths and Limitations

This study is unique in its inclusion of a large, diverse sample of pregnant individuals screened for prenatal cannabis use by self-report and toxicology testing as part of standard prenatal care from 2012 to 2019. We conducted an interrupted time series analysis, which is a rigorous quasi-experimental design. The analysis accounted for pre-RCL trends and included attention to both state RCL passage and local implementation, recognizing that places that allowed vs banned medical retailers before RCL implementation may have different preexisting attitudes toward cannabis and rates of prenatal cannabis use. We also extended prior research by accounting for local policies regarding adult-use retail post-RCL implementation. We also conducted several sensitivity analyses and e-value analyses that supported the primary findings.

However, the sample comprised insured pregnant individuals seeking prenatal care in a state without punitive prenatal substance use policies,^48^ where medical cannabis has been legalized since 1996, and where rates of prenatal cannabis use have been steadily increasing over more than a decade. Results may not generalize to uninsured pregnant individuals, those who do not seek prenatal care, or those in states with different attitudes and criminal justice policies related to prenatal cannabis use. The screening reflects cannabis use during early pregnancy and did not include information about the mode of administration, product strength, or continued use after pregnancy recognition. We standardized rates to adjust for differences in the distribution of age, race and ethnicity, and NDI during the study; however, it is possible that other unmeasured confounders changed over time and might affect results. This study does not account for differences in density of legal retailers, allowance of delivery-only retailers, or access to illicit cannabis. Some pregnant patients living in a jurisdiction that banned cannabis retailers may have been only a short drive from a retailer in another jurisdiction. Finally, while we examined changes during the first approximately 3 years post-RCL passage (2 years post-RCL implementation), we could not assess the longer-term post-RCL implementation trends, particularly because of known changes in prenatal cannabis use during the COVID-19 pandemic.^49^

## Conclusions

In this large, time-series study of pregnancies in Northern California from 2012 to 2019 that were universally screened for cannabis use during early pregnancy by self-report and toxicology testing, the implementation of RCL was associated with an increase in cannabis use during early pregnancy. In local policy analyses, this increase was only found among the subset of individuals living in jurisdictions permitting adult-use retailers. This level change was superimposed on preexisting increases in use concomitant with earlier medical legalization and successive decriminalization measures. Longer-term studies are needed to understand whether trends persist or change further over time.^50^ As legalization spreads across the US, it will be important to test whether local policies that prohibit or limit adult-use retailers moderate changes in prenatal cannabis use associated with state-level legalization.

## Notes

Supplement 1.
eMethods
**eTable 1.** Interrupted Time Series Poisson model results for all three outcomes of prenatal cannabis use, linear trends pre-RCL passage and pre-RCL implementation **eTable 2.** Comparison between those included versus excluded due to missing data on cannabis use during pregnancy (self-report or toxicology test) **eTable 3.** Assessing for changes in rates of prenatal cannabis use before and after RCL passage (before vs. after December 2016), among pregnancies screened during January 1, 2012 to January 31, 2018 **eTable 4.** Main analyses and sensitivity analyses for changes in rates of prenatal cannabis use by self-report or toxicology, accounting for (1) RCL passage and (2) RCL implementation **eTable 5.** Main analyses and sensitivity analyses for changes in rates of prenatal cannabis use by self-report or toxicology, accounting for RCL implementation and cannabis retail policies **eTable 6.** E-values for RCL implementation level change analyses **eFigure 1.** Consort diagram **eFigure 2.** Changes in prenatal cannabis use before and after RCL implementation by: a) toxicology test, and b) self-report, January 1, 2012 to December 31, 2019 **eFigure 3.** Categorization of storefront retail policy corresponding to eFigure 4 analyses, indicating the pre-RCL medical policy and post-RCL any (medical or adult-use) retail policy, among 244 jurisdictions in the Kaiser Permanente Northern California study region **eFigure 4.** Sensitivity analysis of changes in rates of prenatal cannabis use by self-report or toxicology before and after RCL implementation, comparing any post-RCL cannabis retail allowed (medical or adult-use) vs. all cannabis use retail banned **eFigure 5.** Sensitivity analyses of changes in rates of prenatal cannabis use by self-report or toxicology, stratified by pre-RCL medical storefront retail policy and comparing adult-use retail allowed vs banned

Supplement 2. Data Sharing Statement
