# Supplementary material for: Cannabis Use During Early Pregnancy Following Recreational Cannabis Legalization
Source: JAMA Health Forum. 2024 Nov 1;5(11):e243656. doi: 10.1001/jamahealthforum.2024.3656 (PMC11530934; doi:10.1001/jamahealthforum.2024.3656)
Supplement: Supplement 1. — eMethods eTable 1. Interrupted Time Series Poisson model results for all three outcomes of prenatal cannabis use, linear trends pre-RCL passage and pre-RCL implementation eTable 2. Comparison between those included versus excluded due to missing data on cannabis use during pregnancy (self-report or toxicology test) eTable 3. Assessing for changes in rates of prenatal cannabis use before and after RCL passage (before vs. after December 2016), among pregnancies screened during January 1, 2012 to January 31, 2018 eTable 4. Main analyses and sensitivity analyses for changes in rates of prenatal cannabis use by self-report or toxicology, accounting for (1) RCL passage and (2) RCL implementation eTable 5. Main analyses and sensitivity analyses for changes in rates of prenatal cannabis use by self-report or toxicology, accounting for RCL implementation and cannabis retail policies eTable 6. E-values for RCL implementation level change analyses eFigure 1. Consort diagram eFigure 2. Changes in prenatal cannabis use before and after RCL implementation by: a) toxicology test, and b) self-report, January 1, 2012 to December 31, 2019 eFigure 3. Categorization of storefront retail policy corresponding to eFigure 4 analyses, indicating the pre-RCL medical policy and post-RCL any (medical or adult-use) retail policy, among 244 jurisdictions in the Kaiser Permanente Northern California study region eFigure 4. Sensitivity analysis of changes in rates of prenatal cannabis use by self-report or toxicology before and after RCL implementation, comparing any post-RCL cannabis retail allowed (medical or adult-use) vs. all cannabis use retail banned eFigure 5. Sensitivity analyses of changes in rates of prenatal cannabis use by self-report or toxicology, stratified by pre-RCL medical storefront retail policy and comparing adult-use retail allowed vs banned [file jamahealthforum-e243656-s001.pdf]

Supplemental Online Content

Young-Wolff KC, Slama NE, Avalos LA, et al. Cannabis use during early pregnancy following recreational cannabis legalization. *JAMA Health Forum*. 2024;5(10):e243656. doi:10.1001/jamahealthforum.2024.3656

eMethods

eTable 1. Interrupted Time Series Poisson model results for all three outcomes of prenatal cannabis use, linear trends pre-RCL passage and pre-RCL implementation

eTable 2. Comparison between those included versus excluded due to missing data on cannabis use during pregnancy (self-report or toxicology test)

eTable 3. Assessing for changes in rates of prenatal cannabis use before and after RCL passage (before vs. after December 2016), among pregnancies screened during January 1, 2012 to January 31, 2018

eTable 4. Main analyses and sensitivity analyses for changes in rates of prenatal cannabis use by self-report or toxicology, accounting for (1) RCL passage and (2) RCL implementation

eTable 5. Main analyses and sensitivity analyses for changes in rates of prenatal cannabis use by self-report or toxicology, accounting for RCL implementation and cannabis retail policies

eTable 6. E-values for RCL implementation level change analyses

eFigure 1. Consort diagram

eFigure 2. Changes in prenatal cannabis use before and after RCL implementation by: a) toxicology test, and b) self-report, January 1, 2012 to December 31, 2019

eFigure 3. Categorization of storefront retail policy corresponding to eFigure 4 analyses, indicating the pre-RCL medical policy and post-RCL any (medical or adult-use) retail policy, among 244 jurisdictions in the Kaiser Permanente Northern California study region

eFigure 4. Sensitivity analysis of changes in rates of prenatal cannabis use by self-report or toxicology before and after RCL implementation, comparing any post-RCL cannabis retail allowed (medical or adult-use) vs. all cannabis use retail banned

eFigure 5. Sensitivity analyses of changes in rates of prenatal cannabis use by self-report or toxicology, stratified by pre-RCL medical storefront retail policy and comparing adult-use retail allowed vs banned

This supplemental material has been provided by the authors to give readers additional information about their work.

© 2024 Young-Wolff KC et al. *JAMA Health Forum.*

eMethods

Urine toxicology test details. Cannabis urine toxicology tests were performed on a Beckman Coulter AU680 chemistry analyzer using the Emit II Plus Cannabinoid Assay from Siemens with a cutoff of 45ng/mL. Confirmatory testing for the presence of the cannabis metabolite, 11-nor-9-carboxy-delta 9- THC, was performed by liquid chromatography-tandem mass spectrometry for all positive immunoassay results. The confirmation test methodology was LC-MS/MS on a triple quadrupole system with a cutoff for positivity of 15ng/mL.

Interrupted Time Series Model Corresponding to main analyses shown in Figure 1

$$Y_t = \exp\,(\beta_0 + \beta_1 T + \beta_2 X_t + \beta_3 T_A)$$

Where Y_t is the observed outcome count at time T, T is the time elapsed since the study start with months as the unit, T_A is the time elapsed after the intervention began with months as the unit, and X_t is a binary variable indicating the post-intervention period (coded 1 for post-intervention, 0 for pre-intervention). The estimated parameters are β_0 the intercept, β_1 the slope representing the trend over time, β_2 the level change following the intervention, β_3 represents the slope change following the intervention. Using this model, the pre-intervention slope is represented by β_1 and the post-intervention slope is represented by $\beta_1 + \beta_3$. The model also included an offset comprised of the log of the standardized population for each month.

© 2024 Young-Wolff KC et al. *JAMA Health Forum.*

eTable 1. Interrupted Time Series Poisson model results for all three outcomes of prenatal cannabis use, linear trends pre-RCL passage and pre-RCL implementation

Outcome	Annual trend pre-RCL passage Jan 2012 – Nov 2016	Test for non-linearity using quadratic term for time	Annual trend pre-RCL implementation, Jan 2012 – Jan 2018
	**RR (95% CI)**	**P-value**	**RR (95% CI)**
**Self-report or toxicology**	1.079 (1.064-1.093)	0.83	1.089 (1.079-1.099)
**Toxicology**	1.083 (1.068-1.099)	0.36	1.087 (1.076-1.097)
**Self-report**	1.070 (1.049-1.092)	0.16	1.087 (1.072-1.103)

Notes: CI = confidence interval. RCL = Recreational Cannabis Legalization. RR = relative rate.

© 2024 Young-Wolff KC et al. *JAMA Health Forum.*

eTable 2. Comparison between those included versus excluded due to missing data on cannabis use during pregnancy (self-report or toxicology test)

	N (%)		
Characteristics	Excluded N=21,581 (6.7%)	Included N=300,993 (93.3%)	Standardized difference[a]
Age, years			0.19
< 24	2692 (12.5)	44,437 (14.8)	
25-34	12,449 (57.7)	189,259 (62.9)	
35+	6440 (29.8)	67,297 (22.4)	
Race and ethnicity			0.04
Hispanic	5292 (24.5)	78,304 (26.0)	
Non-Hispanic			
Asian	5708 (26.5)	77,799 (25.9)	
Black	1423 (6.6)	19,330 (6.4)	
White	8196 (38.0)	113,324 (37.6)	
Other/multiracial/unknown	962 (4.5)	12,236 (4.1)	
Medicaid insurance	1262 (5.9)	19,899 (6.6)	0.03
Neighborhood deprivation index			0.19
Quartile 1 (least deprived)	5589 (25.9)	71,667 (23.8)	
Quartile 2	5023 (23.3)	71,050 (23.6)	
Quartile 3	4735 (21.9)	70,793 (23.6)	
Quartile 4 (most deprived)	4423 (20.5)	71,598 (23.8)	
Unknown	1811 (8.3)	15,885 (5.3)	

Notes. Other race includes American Indian, Alaska Native, Hawaiian Islander, and Pacific Islander. Percentages may not add to 100 due to rounding.

[a]Standardized difference = difference in proportions divided by standard error; imbalance defined as absolute value greater than 0.20 (small effect size).

© 2024 Young-Wolff KC et al. *JAMA Health Forum.*

eTable 3. Assessing for changes in rates of prenatal cannabis use before and after RCL passage (before vs. after December 2016), among pregnancies screened during January 1, 2012 to January 31, 2018

	Annual trend pre-RCL passage		RCL passage level change		Annual trend post-RCL passage	
**Outcome**	**RR (95% CI)**	**Slope P-value**	**RR (95% CI)**	**Level change P-value**	**RR (95% CI)**	**Pre vs Post slope change P-value**
**Self-report or toxicology**	1.079 (1.065-1.094)	<.001	1.030 (0.956-1.109)	.44	1.120 (1.022-1.228)	.42
**Toxicology**	1.089 (1.069-1.099)	<.001	1.023 (0.945-1.109)	.57	1.076 (0.974-1.189)	.89
**Self-report**	1.070 (1.049-1.091)	<.001	1.019 (0.910-1.142)	.74	1.191 (1.036-1.369)	.14

Notes: CI = confidence interval. RCL = Recreational Cannabis Legalization. RR = relative rate. A one-month lag was used to define pregnancies screened pre vs. post-RCL passage in the monthly screening data.

© 2024 Young-Wolff KC et al. *JAMA Health Forum.*

eTable 4. Main analyses and sensitivity analyses for changes in rates of prenatal cannabis use by self-report or toxicology, accounting for (1) RCL passage and (2) RCL implementation

**Figure 1a analysis**		
**Effect**	**RR (95% CI)**	**Pre vs Post slope change p-value**
Pre-RCL passage annual trend	1.079 (1.065 - 1.093)	--
RCL passage level change	1.030 (0.956 - 1.109)	--
Post-RCL passage annual trend	1.120 (1.022 - 1.228)	0.42

**Figure 1a seasonally adjusted sensitivity analysis**		
**Effect**	**RR (95% CI)**	**Pre vs Post slope change p-value**
Pre-RCL passage annual trend	1.080 (1.066 - 1.094)	--
RCL passage level change	1.018 (0.943 - 1.099)	--
Post-RCL passage annual trend	1.142 (1.038 - 1.255)	0.25

**Figure 1b analysis**		
**Effect**	**RR (95% CI)**	**Pre vs Post slope change p-value**
Pre-RCL implementation annual trend	1.089 (1.079 - 1.099)	--
RCL implementation level change	1.097 (1.035 - 1.163)	--
Post-RCL implementation annual trend	1.019 (0.975 - 1.064)	0.004

**Figure 1b seasonally adjusted sensitivity analysis**		
**Effect**	**RR (95% CI)**	**Pre vs Post slope change p-value**
Pre-RCL implementation annual trend	1.090 (1.080 - 1.101)	--
RCL implementation level change	1.078 (1.017 - 1.144)	--
Post-RCL implementation annual trend	1.032 (0.988 - 1.079)	.02

**Figure 1b Feb 2018 sensitivity analysis**		
**Effect**	**RR (95% CI)**	**Pre vs Post slope change p-value**
Pre-RCL implementation annual trend	1.089 (1.079 - 1.099)	--
RCL implementation level change – Feb 2018	1.221 (1.092 - 1.364)	--
RCL implementation level change – March 2018 and later	1.066 (1.005 - 1.130)	--
Post-RCL implementation annual trend	1.038 (0.991 - 1.087)	0.048

Notes. CI = confidence interval. RCL = Recreational Cannabis Legalization. RR = relative rate. Rates are standardized to age, race and ethnicity, and neighborhood deprivation index of the pregnancies in the study sample during 2019. Prenatal cannabis use was based on a positive urine toxicology test or self-report conducted as part of standard prenatal care (typically at ~8-10 weeks gestation).

© 2024 Young-Wolff KC et al. *JAMA Health Forum.*

eTable 5. Main analyses and sensitivity analyses for changes in rates of prenatal cannabis use by self-report or toxicology, accounting for RCL implementation and cannabis retail policies

**Figure 2 analyses**	**Retail banned[a]**		**Retail allowed[a]**		
**Effect**	**RR (95% CI)**	**Pre vs Post slope change p-value**	**RR (95% CI)**	**Pre vs Post slope change p-value**	**Ban*allow interaction p-value**
Pre-RCL implementation annual trend	1.107 (1.091-1.123)	--	1.070 (1.055-1.085)	--	N/A (stratified models)
RCL implementation level change	1.074 (0.984-1.173)	--	1.124 (1.032-1.224)	--	N/A (stratified models)
Post-RCL implementation annual trend	1.011 (0.946-1.081)	0.01	1.030 (0.967-1.098)	0.26	N/A (stratified models)
**Figure 4 analyses**	**Retail banned[b]**		**Retail allowed[b]**		
**Effect**	**RR (95% CI)**	**Pre vs Post slope change p-value**	**RR (95% CI)**	**Pre vs Post slope change p-value**	**Ban*allow interaction p-value**
Pre-RCL implementation annual trend	1.107 (1.091 - 1.122)	--	1.070 (1.054 - 1.086)	--	0.001
RCL implementation level change	1.014 (0.933 - 1.103)	--	1.210 (1.100 - 1.331)	--	0.001
Post-RCL implementation annual trend	1.061 (0.993 - 1.135)	0.21	1.001 (0.933 - 1.072)	0.06	0.60
**Figure 4 Feb 2018 sensitivity analysis**	**Retail banned[b]**		**Retail allowed[b]**		
**Effect**	**RR (95% CI)**	**Pre vs Post slope change p-value**	**RR (95% CI)**	**Pre vs Post slope change p-value**	**Ban*allow interaction p-value**
Pre-RCL implementation annual trend	1.107 (1.092 - 1.122)	--	1.070 (1.055 - 1.085)	--	0.001
RCL implementation level change – Feb 2018	1.123 (0.968 - 1.303)	--	1.470 (1.200 - 1.801)	--	<0.001
RCL implementation level change – March 2018 and later	0.985 (0.905 - 1.073)	--	1.165 (1.059 - 1.282)	--	0.008

© 2024 Young-Wolff KC et al. *JAMA Health Forum.*

Post-RCL implementation annual trend	1.084 (1.009 - 1.163)	0.55	1.026 (0.954 - 1.103)	0.26	0.99

**eFigure 4 analyses**	**Retail banned[c]**		**Retail allowed[c]**		
**Effect**	**RR (95% CI)**	**Pre vs Post slope change p-value**	**RR (95% CI)**	**Pre vs Post slope change p-value**	**Ban*allow interaction p-value**
Pre-RCL implementation annual trend	1.107 (1.091 - 1.122)	--	1.070 (1.055 - 1.085)	--	0.001
RCL implementation level change	1.025 (0.939 - 1.118)	--	1.171 (1.074 - 1.276)	--	0.01
Post-RCL implementation annual trend	1.054 (0.984 - 1.129)	0.16	1.001 (0.957 - 1.086)	0.16	0.99

Notes. CI = confidence interval. RCL = Recreational Cannabis Legalization. RR = relative rate. Rates are standardized to age, race and ethnicity, and neighborhood deprivation index of the pregnancies in the study sample during 2019. Prenatal cannabis use was based on a positive urine toxicology test or self-report conducted as part of standard prenatal care (typically at ~8-10 weeks gestation).

[a] Models are stratified by the pre-RCL medical storefront retail policy of each pregnancy (medical allowed vs banned).

[b] Retail policies account for medical retail policy (medical allowed vs. banned) in the pre-RCL implementation period and adult-use retail policy (adult-use allowed vs. banned) in the post-RCL implementation period.

[c] Retail policies account for medical retail policy (medical allowed vs. banned) in the pre-RCL implementation period and medical or adult-use retail policy (medical or adult-use allowed vs. both banned) in the post-RCL implementation period.

© 2024 Young-Wolff KC et al. *JAMA Health Forum.*

eTable 6. E-values for RCL implementation level change analyses

RCL implementation level change	RR (95% CI) reported in paper	e-value for RR	e-value for lower bound of 95% CI
Overall			
Main analysis (Figure 1b)	1.097 (1.035 - 1.163)	1.423	1.225
Toxicology test (eFigure 2a)	1.070 (1.005 - 1.138)	1.344	1.076
Self-report (eFigure 2b)	1.209 (1.109 - 1.319)	1.712	1.457
Stratified by pre-RCL medical policy (Figure 2)			
Medical allowed	1.124 (1.032 - 1.224)	1.497	1.214
Post-RCL adult-use policy (Figure 4)			
Adult-use allowed	1.210 (1.100 - 1.331)	1.714	1.432

Notes. CI = confidence interval. RCL = Recreational Cannabis Legalization. RR = relative rate.

© 2024 Young-Wolff KC et al. *JAMA Health Forum.*

eFigure 1. Consort diagram

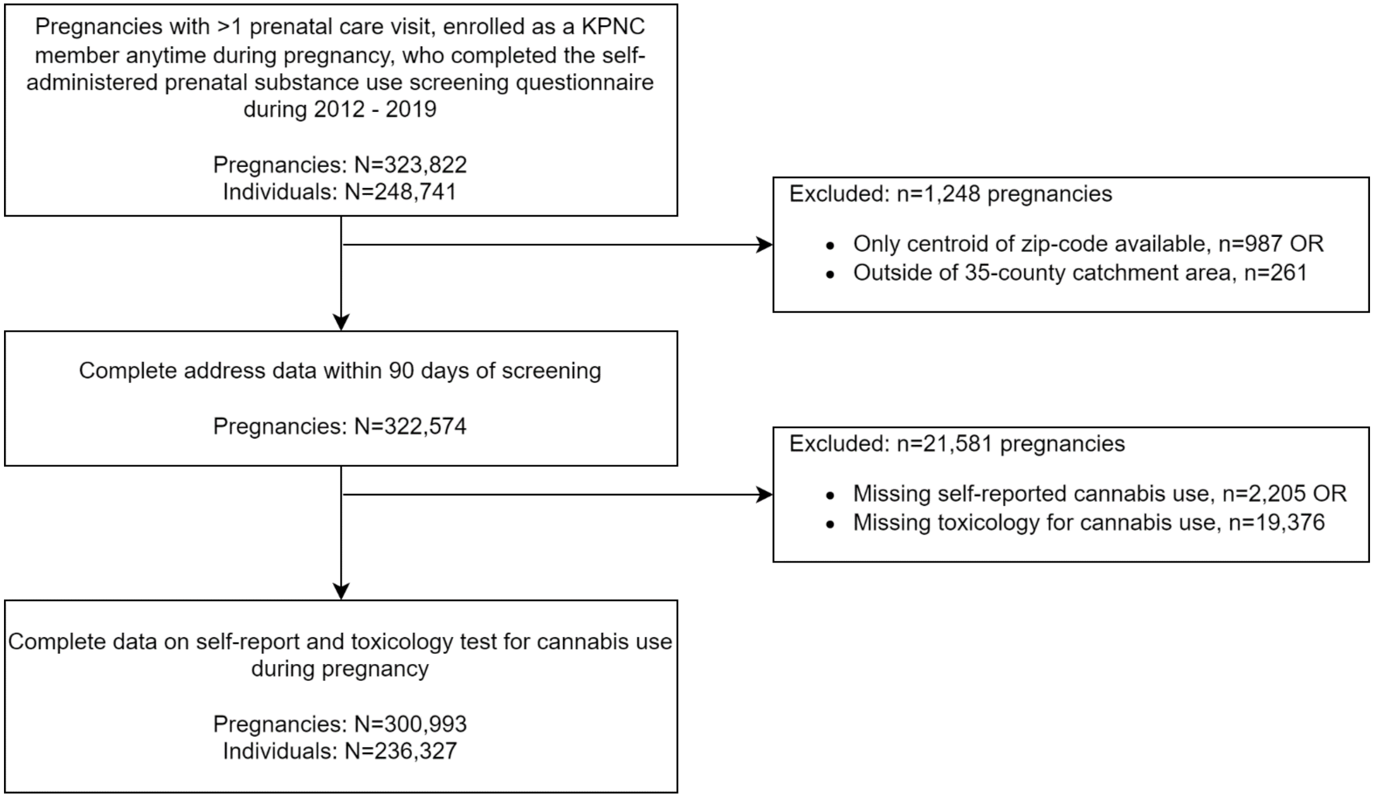

Notes: KPNC = Kaiser Permanente Northern California.

© 2024 Young-Wolff KC et al. *JAMA Health Forum.*

eFigure 2. Changes in prenatal cannabis use before and after RCL implementation by: a) toxicology test, and b) self-report, January 1, 2012 to December 31, 2019

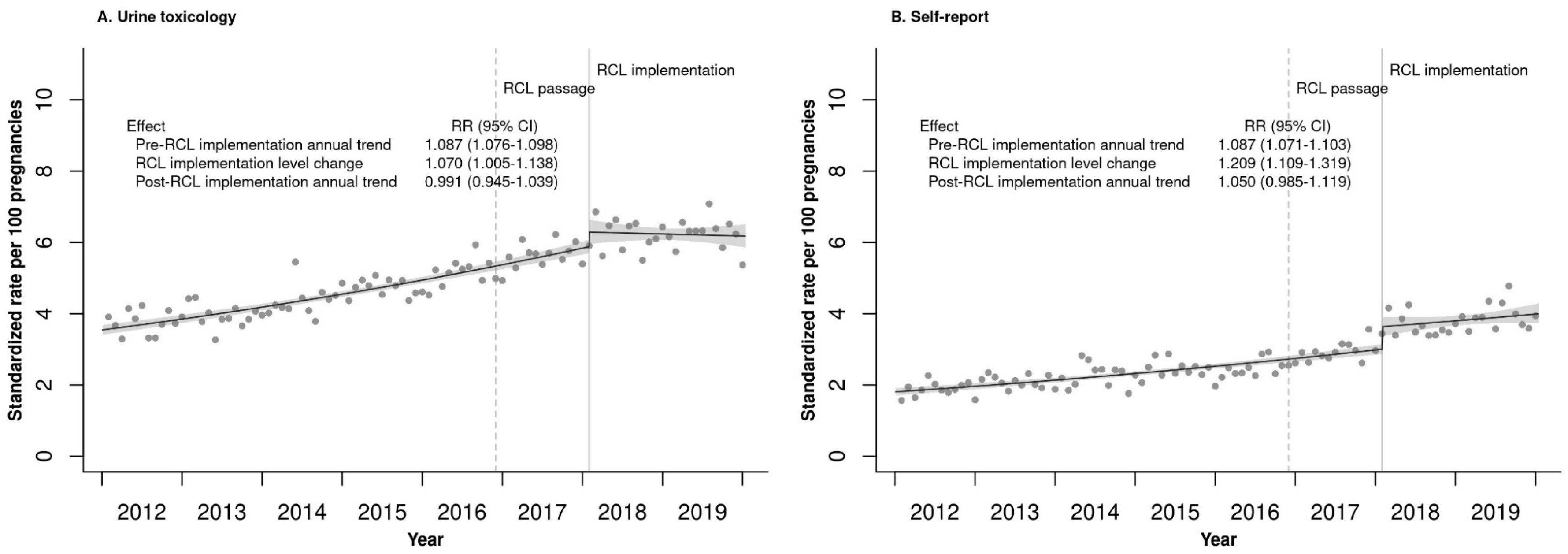

Notes. CI = confidence interval. RCL = Recreational Cannabis Legalization. RR = relative rate. Rates are standardized to age, race and ethnicity, and neighborhood deprivation index of the pregnancies in the overall study sample in year 2019. The lines show the fitted regression of the interrupted time series model and the dots show the monthly standardized rates. Prenatal cannabis use was based on: a) a positive urine toxicology test, and b) self-report conducted as part of standard screening done at entrance to prenatal care (typically at ~8-10 weeks gestation).

© 2024 Young-Wolff KC et al. *JAMA Health Forum.*

eFigure 3. Categorization of storefront retail policy corresponding to eFigure 4 analyses, indicating the pre-RCL medical policy (allowed, green; banned, yellow) and post-RCL any (medical or adult-use) retail policy (allowed, blue; banned, red), among 244 jurisdictions in the Kaiser Permanente Northern California study region

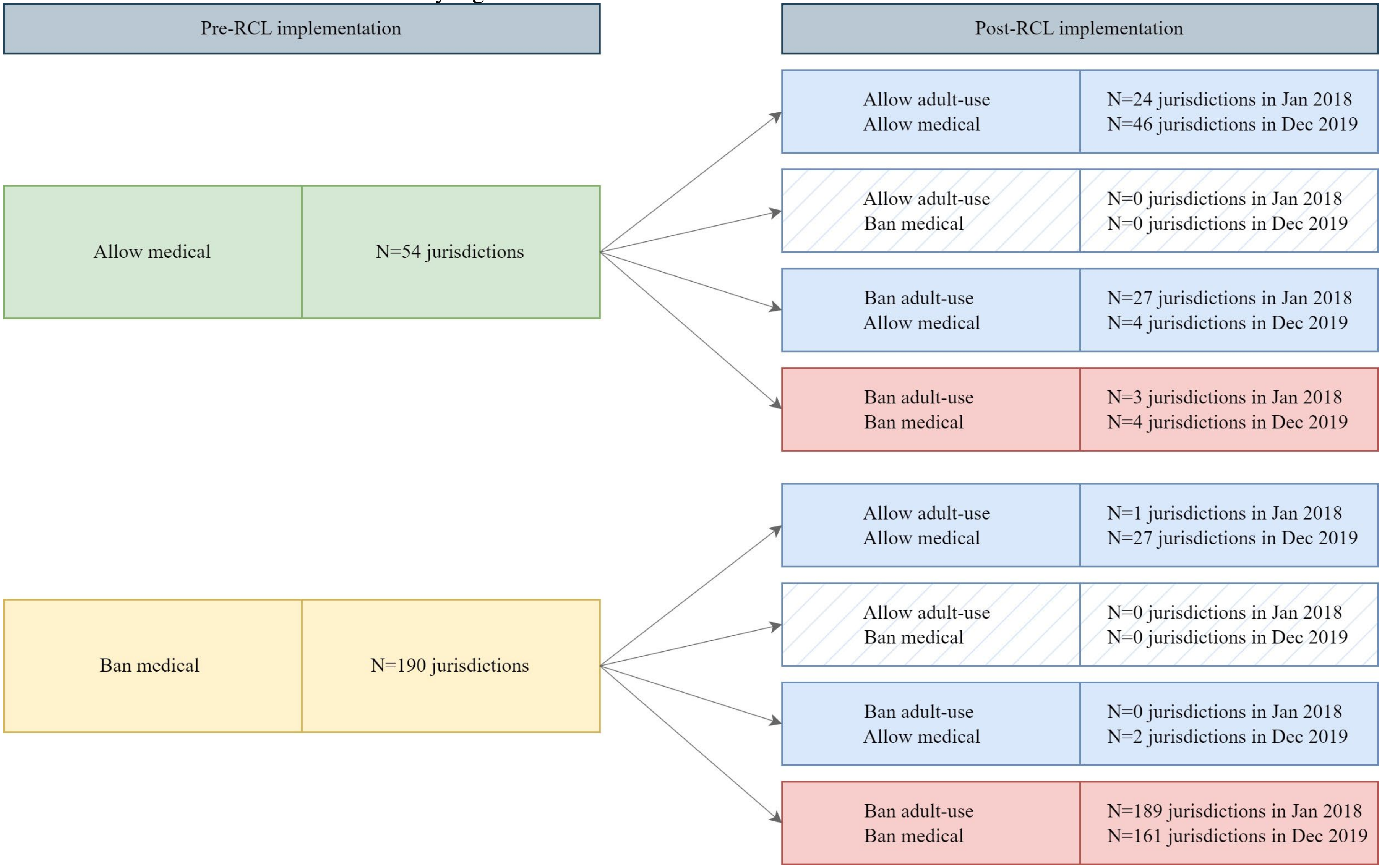

Notes. RCL = recreational cannabis legalization. In the period pre-RCL implementation, pregnancies were grouped based on whether medical retail was allowed (green) or banned (yellow) in their jurisdiction. In the period post-RCL implementation, pregnancies were grouped based on whether any retailer (adult-use or medical)

© 2024 Young-Wolff KC et al. *JAMA Health Forum.*

was allowed (blue) or banned (red) in each jurisdiction at the time of their screening (corresponds to analysis in eFigure 4). Initially, 217 (88.9%) of the 244 jurisdictions had the same policy for medical as for adult-use storefront retailers (e.g., both allowed or both banned). By the end of the study period, 51 (20.9%) jurisdictions changed policies and 238 (97%) of jurisdictions enacted the same policy for medical and adult-use storefront retailers.

© 2024 Young-Wolff KC et al. *JAMA Health Forum.*

eFigure 4. Sensitivity analysis of changes in rates of prenatal cannabis use by self-report or toxicology before and after RCL implementation, comparing any post-RCL cannabis retail allowed (medical or adult-use) vs. all cannabis use retail banned

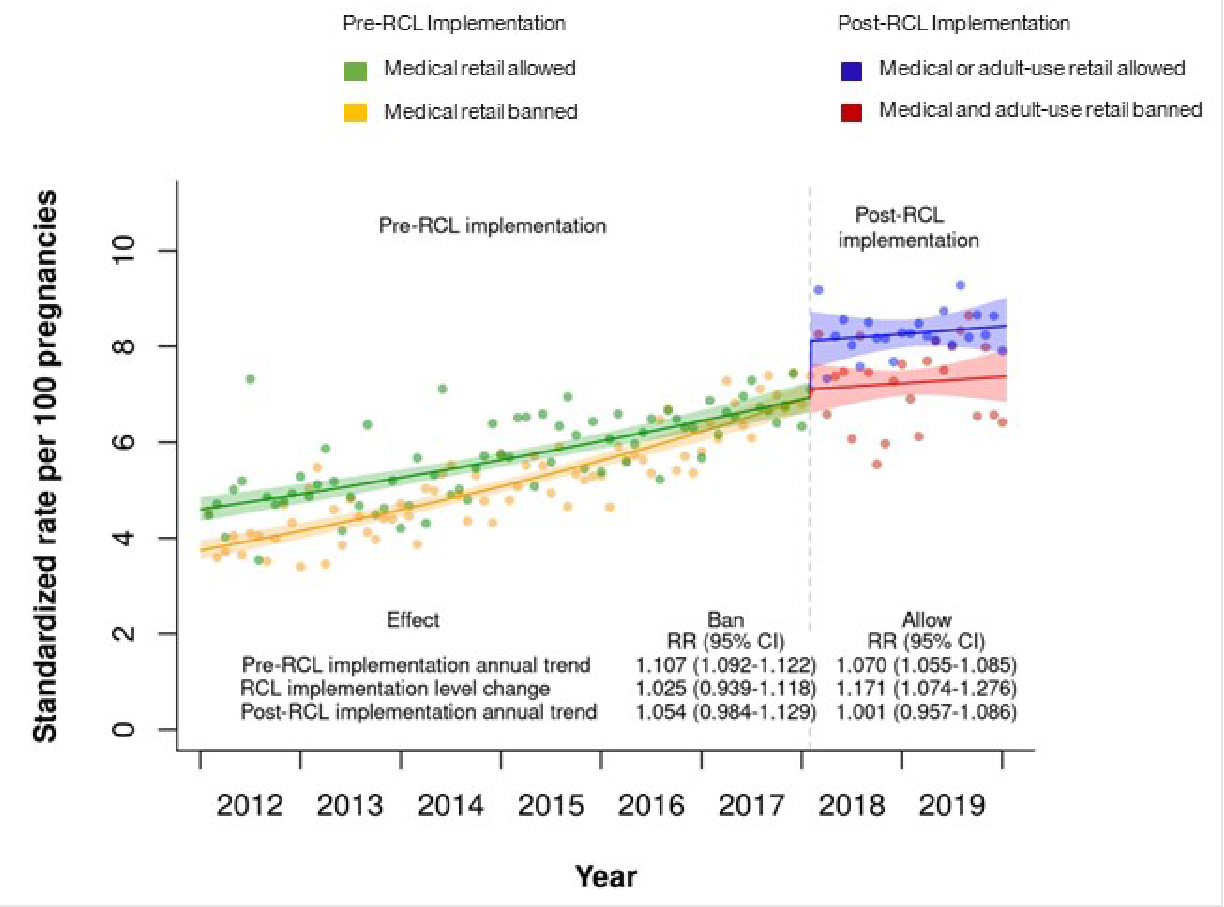

Notes. CI = confidence interval. RCL = Recreational Cannabis Legalization. RR = relative rate. Rates are standardized to age, race and ethnicity and neighborhood deprivation index of the pregnancies in the overall study sample during 2019. The lines show the fitted regression of the interrupted time series model and the dots show the monthly standardized rates. Prenatal cannabis use was based on a positive urine toxicology test or self-report conducted as part of standard prenatal care (typically at ~8-10 weeks gestation).

© 2024 Young-Wolff KC et al. *JAMA Health Forum.*

eFigure 5. Sensitivity analyses of changes in rates of prenatal cannabis use by self-report or toxicology, stratified by pre-RCL medical storefront retail policy and comparing adult-use retail allowed vs banned

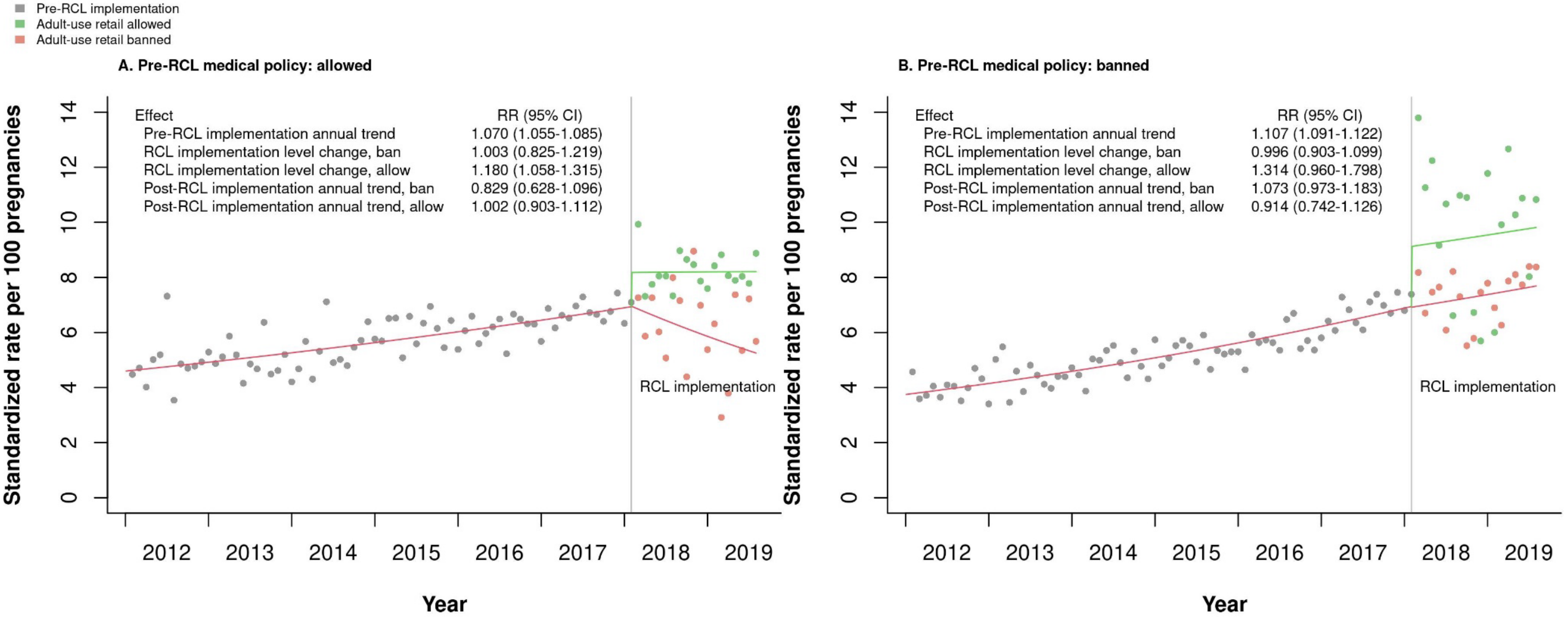

Notes. CI = confidence interval. RCL = Recreational Cannabis Legalization. RR = relative rate. Rates are standardized to age, race and ethnicity and neighborhood deprivation index of the pregnancies in the overall study sample during 2019. The lines show the fitted regression of the interrupted time series model and the dots show the monthly standardized rates. Prenatal cannabis use was based on a positive urine toxicology test or self-report conducted as part of standard prenatal care (typically ~8-10 weeks gestation). Retail policies account for medical retail policy (medical allowed vs. banned) in the pre-RCL implementation and adult-use retail policy (adult-use allowed vs. banned) in the post-RCL implementation period. Due to sparsity, these analyses were truncated at July 2019.

© 2024 Young-Wolff KC et al. *JAMA Health Forum.*
