# Supplementary material for: Cannabis Use During Early Pregnancy Following Recreational Cannabis Legalization
Source: JAMA Health Forum. 2024 Nov 1;5(11):e243656. doi: 10.1001/jamahealthforum.2024.3656 (PMC11530934; doi:10.1001/jamahealthforum.2024.3656)
Supplement: Supplement 2. — Data Sharing Statement [file jamahealthforum-e243656-s002.pdf]

Data Sharing Statement

Young-Wolff. Cannabis Use During Early Pregnancy Following Recreational Cannabis Legalization. *JAMA Health Forum*. Published November 01, 2024. doi:10.1001/jamahealthforum.2024.3656

Data
Data available: No
